# Renoprotective effect of thymoquinone against rhabdomyolysis-induced acute kidney injury in the rat model

**DOI:** 10.22038/IJBMS.2023.72797.15838

**Published:** 2024

**Authors:** Arezoo Hosseini, Soghra Mehri, Tahereh Aminifard, Mahboobeh Ghasemzadeh Rahbardar, Sadaf Nouripor, Abolfazl Khajavi rad, Amirhossein Jafarian, Hossein Hosseinzadeh

**Affiliations:** 1 Department of Pharmacodynamics and Toxicology, School of Pharmacy, Mashhad University of Medical Sciences, Mashhad, Iran; 2 Pharmaceutical Research Center, Pharmaceutical Technology Institute, Mashhad University of Medical Sciences, Mashhad, Iran; 3 Department of Physiology, School of Medicine, Mashhad University of Medical Sciences, Mashhad, Iran; 4 Department of Pathology, Ghaem Hospital, Mashhad University of Medical Sciences, Mashhad, Iran

**Keywords:** Creatinine, Glycerol, Inflammation, Myoglobin, Nigella sativa

## Abstract

**Objective(s):**

Rhabdomyolysis leads to the release of myoglobin, sarcoplasmic proteins, and electrolytes into the blood circulation causing acute kidney injury (AKI). Thymoquinone, a natural compound found in *Nigella sativa* seeds, has antioxidant and anti-inflammatory effects. This investigation assessed the renoprotective effect of thymoquinone on rhabdomyolysis-induced AKI in rats.

**Materials and Methods:**

Male Wistar rats were categorized into six groups (n = 6): 1. Control: (normal saline), 2. Glycerol (50 ml/kg, single dose, IM), 3–5: Glycerol + thymoquinone (1, 2.5 and 5 mg/kg, 4 days, IP), 6. Thymoquinone (5 mg/kg). On day 5, serum and kidney tissue were isolated and the amounts of serum creatinine and blood urea nitrogen (BUN), renal malondialdehyde (MDA), glutathione (GSH.), tumor necrosis factor-alpha (TNF-α), neutrophil gelatinase-associated lipocalin (NGAL), and pathological changes were evaluated.

**Results:**

Glycerol increased creatinine, BUN, MDA, TNF-α, and NGAL levels. It decreased GSH amounts and caused renal tubular necrosis, glomerular atrophy, and myoglobin cast in kidney tissue. Co-administration of glycerol and thymoquinone reduced creatinine, BUN, histopathological alterations, and MDA levels, and enhanced GSH amounts. Administration of glycerol and thymoquinone (5 mg/kg) had no significant effect on TNF-α amount but decreased NGAL protein levels. The administration of thymoquinone (5 mg/kg) alone did not display a significant difference from the control group.

**Conclusion:**

Rhabdomyolysis from glycerol injection in rats can cause kidney damage. Thymoquinone may attenuate renal dysfunction and oxidative stress. However, the TNF-α level was not significantly affected. Further studies are needed to explore the potential therapeutic effects of thymoquinone in managing AKI.

## Introduction

Myoglobin and sarcoplasmic proteins are released from damaged muscles as a result of rhabdomyolysis, which is characterized by acute and severe muscular injury. Not only that, but also electrolytes release into the extracellular space and circulation, developing symptoms such as pain, weakness, and/or edema. Rhabdomyolysis might typically occur after the trauma but can also result from various clinical situations, such as sudden temperature changes, prolonged muscle compression, and intense physical activity (1). Acute kidney injury (AKI) is only one of the many signs of rhabdomyolysis, which can occur in 10–40% of rhabdomyolysis patients (2). Due to the redox cycling of the heme group in rhabdomyolysis-induced AKI, the heme component of myoglobin can trigger lipid peroxidation and cause kidney damage (3). Activation of pro-inflammatory pathways has also been linked to rhabdomyolysis-induced AKI, which leads to damage to the tubular epithelium and stimulates fibrosis. Besides, neutrophil gelatinase-associated lipocalin (NGAL), a stress protein belonging to the lipocalin superfamily, is recognized as an early diagnostic biomarker for AKI. The absence of effective treatment strategies to inhibit or speed up the recovery from AKI has prompted a significant research effort to identify and develop novel treatment options for this condition (4).

The glycerol-induced AKI model is a widely utilized animal model for exploring the pathogenesis of AKI and identifying potential therapeutic targets (5). The intense breakdown of skeletal muscle tissue releases myoglobin, which can exceed the capacity of plasma protein-binding and subsequently undergo glomerular filtration and tubular reabsorption. Myoglobin increases in the renal tubules and ultimately can cause renal failure (6). Previous studies showed that natural antioxidants can alleviate or prevent AKI-associated tissue damage. This is hypothesized to happen as a result of excessive free radical scavenging and lipid peroxidation suppression (4, 7, 8).

*Nigella sativa*, a seed recognized for its unique flavor, is a member of the Ranunculaceae family and commonly referred to as black seed or black cumin. In addition to its culinary applications as a seasoning and food preservative, this seed᾿s potential health-improving abilities have been considered (9). *N. sativa* has been utilized for various health conditions, including ischemia (10), renal disorders (11), rheumatoid arthritis (12), metabolic disorder (13), seizure (14), and memory dysfunction (15). Thymoquinone, thymohydroquinone, alpha-herein, thymol, nigellimine-N-oxide, carvacrol, dithymoquinone, nigellicine, and nigellidine as important constituents, are responsible for the biological activity of *N. sativa* (9). Thymoquinone and black cumin seed, utilized as oils, extracts, and powders, have been shown in preclinical studies to be protective against kidney damage brought on by ischemia (16, 17), as well as chemotherapy drugs used to treat cancer, such as methotrexate and cisplatin (18). In addition to its immune-modulating ability, it has exceptional anti-oxidant (19, 20)and anti-inflammatory (20, 21) properties. 

This study examined the effect of thymoquinone on rhabdomyolysis-induced AKI caused by glycerol injection, based on the ameliorative effects of thymoquinone and the underlying pathways involved in the pathophysiology of AKI. To the best of our knowledge, this is the first investigation in medical literature to evaluate the effectiveness of thymoquinone (1, 2.5, 5 mg/kg, 4 days, IP) in preventing the development of AKI in rats.

## Materials and Methods

***Chemicals***

The chemicals utilized in this research were acquired from various sources. Potassium chloride (KCl), thiobarbituric acid (TBA), and phosphoric acid were purchased from Merck Company, Germany. Sigma Company, Germany, provided trichloroacetic acid (TCA), tetrabutylammonium, and thymoquinone, while polyvinylidene fluoride (PVDF) membrane was obtained from Millipore (Billerica, MA, USA). In addition, 5,5′-Dithiobis 2-nitrobenzoic acid (DTNB) was synthesized by Sigma Company located in the USA.

***Experimental design***

Thirty-six adult male Wistar rats weighing between 200 and 250 g were acquired from the animal house at the School of Pharmacy, Mashhad University of Medical Sciences, Iran. The animals were kept under controlled conditions with 12-hr light-dark cycles, a temperature range of 21–24 °C, and humidity levels between 40–60%. Mashhad University of Medical Sciences ethical committee (ethical number: IR.MUMS.PHARMACY.REC.1400.105) rules and procedures were followed for using rats in this work. Following a two-day acclimation period, six groups of rats were randomly assigned to different groups (n=6), as indicated in Table 1. AKI was induced by intramuscular injections of 50% glycerol (10 ml/kg), diluted in saline (0.9% NaCl), into the hind limbs of the rats. Before the glycerol injection, the rats underwent 16 hr of dehydration. The control group received vehicle (normal saline) injections through the same route (22). Thymoquinone was dissolved in dimethylsulfoxide (DMSO) (0.1% v/v) and saline solution for intraperitoneal injections. The chosen dose of thymoquinone was based on a previous study (22). 

***Sample collection***

Following the treatment period, the rats were euthanized, and their serum and kidney tissues were collected for biochemical and pathological analysis. Anesthesia was administered to the rats by intraperitoneal injection of ketamine and xylazine at a dose of 5 mg/kg and 50 mg/kg, respectively (23). The right kidneys were promptly frozen in liquid nitrogen and stored at a temperature of -80 ^°^C until analysis, while the left kidneys were preserved in 10% formalin for subsequent pathological examination.

***Evaluating the renal function***

The levels of serum creatinine and blood urea nitrogen (BUN) were determined for the assessment of kidney function.

***Histopathological analysis***

Hematoxylin and eosin (H-E) staining were utilized to stain paraffin blocks of the kidney tissues, which were sectioned at 2 µm intervals (24). An image analyzer equipped with a microscope (Olympus BX-51Tokyo, Japan) was utilized to examine the images. Renal damage was semi-quantitatively scored based on the percentage of damaged renal area, tubular necrosis, protein cast formation, and glomerular atrophy as follows: (-). Normal, (+). mild damage (5–25% of tubules affected), (**++**). moderate damage (26–50% of tubules affected), (**+++**). severe damage (50–75% of tubules affected), and (**++++**). extensive damage (over 75% of tubules affected).

***Lipid peroxidation assay***

Malondialdehyde (MDA) levels can be used as indicators of lipid peroxidation, and an increase in the levels suggests elevated lipid peroxidation. In an acidic environment, MDA reacts with TBA to form a pink-colored complex with maximum absorption at 532 nm (25). First, a 10% homogenate was prepared in 1.15% cold KCl. Next, 0.5 ml of this homogenate was mixed with 1 ml of 0.6% TBA solution and 3 ml of 1% phosphoric acid. The tubes containing the mixture were then placed in boiling water for 45 min. After cooling, the mixture was vortexed with 4 ml of n-butanol for one minute to extract the colored complex. The resulting mixture was centrifuged for 10 min at 3000 rpm, and the organic phase of the supernatant was transferred to new tubes. The absorbance of each sample was measured at 532 nm. Finally, the MDA concentration was expressed as nmol/g tissue (26).

***Determination of glutathione (GSH) content***

The assay relies on the interaction between free sulfhydryl groups and the DTNB reagent under alkaline conditions, resulting in the formation of a colored complex that exhibits peak absorbance at 412 nm (27). 

To conduct the analysis, the tissue samples were dissected and a 10% homogenate was prepared using a phosphate buffer solution at pH 7.4. The homogenate was then mixed with 10% TCA in a 1:1 ratio and subjected to centrifugation at 2500 g for 10 min. The upper phase was isolated and blended with 2 ml of phosphate buffer at pH 8. Next, 0.5 ml of 0.04% DTNB reagent was added to each sample, and the absorbance of the resulting mixture was measured at a wavelength of 412 nm. The concentration of GSH in each sample was determined and expressed as nmol/g tissue (28).

***Western blotting test***

The tissue samples were placed and defrosted in a lysis buffer containing 10 mM sodium azide, 1 mM sodium orthovanadate (Na_3_VO_4_), 1 mM phenylmethylsulfonyl fluoride, 10 mM glycerophosphate, 50 mM Tris-HCl (pH 7.4), 2 mM egtazic acid (EGTA), 2 mM ethylenediaminetetraacetic acid (EDTA), 0.2% W/V sodium deoxycholate, and a complete protease (Sigma, Mannheim, Germany). Then the homogenate was centrifuged at 10,000 g for 10 min at 4 °C after being subjected to three 10 sec bursts of high-intensity sonication on ice with a 10 sec cooling time in between. The protein content was calculated using the Bradford test kit (BioRad, USA). Each sample was boiled, aliquoted, and stored at -80 °C by being mixed 1:1 V/V with 2X SDS blue buffer (29). Samples were loaded for tumor necrosis factor-alpha (TNF-α) and NGAL, electrophoresed on a 12% gel, and transferred to a PVDF membrane (BioRad, USA). The membranes were blocked for 2 hr with 5% nonfat milk powder (skimmed milk) at room temperature. After that, the membranes underwent three rinses in TBST (tris-buffered saline and tween 20). They were then incubated on a rocker for 16 to 18 hr at 4 °C with rabbit polyclonal anti-serum against TNF-α antibody (Cell signaling, #3707, 1: 1,000), and rabbit monoclonal anti-serum against NGAL (Abcam#216462, 1: 1,000), or incubated for 2 hr in room temperature with mouse polyclonal anti-β-actin antibodies (Cell signaling #3700, 1: 1,000). Following three washes with TBST, the membranes were incubated with rabbit or mouse horseradish peroxidase-conjugated anti-IgG (Cell Signaling #7074, 1:2000; Cell Signaling #7076, 1:2000, respectively) for 2 hr. The peroxidase-coated bands were visualized using enhanced chemiluminescence. Utilizing the Alliance 4.7 Gel Doc program (UK) and UV Tec software (UK), the integrated optical densities of the bands were measured, and densitometric analysis of the protein bands was carried out. The protein concentrations were adjusted to the appropriate bands of the β-actin control protein.

***Statistical analysis***

The results were presented as mean ± standard deviation (SD). Statistical analysis was performed using GraphPad Prism 8.0 (Software Inc., San Diego, CA, USA). For MDA and GSH levels, as well as serum BUN and creatinine, the data were expressed as mean ± SD, and comparisons between different groups were made using a one-way ANOVA test with Tukey-Kramer post-test. A *P*-value greater than 0.05 was considered statistically significant.

## Results

***Effects of thymoquinone on glycerol-induced kidney dysfunction***

**Figure 1:**
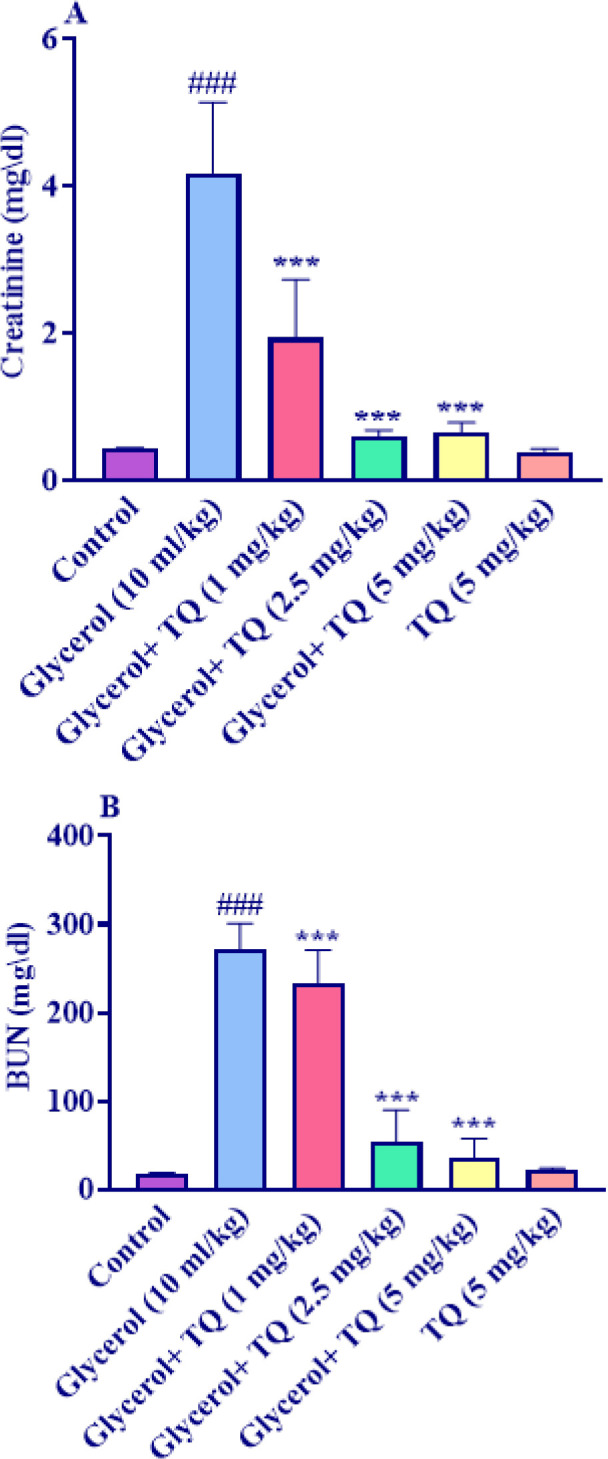
Effect of glycerol 50% (10 ml/kg) and thymoquinone (1, 2.5, and 5 mg/kg) on serum in Wistar rats A: Creatinine and B: BUN levels

According to the findings, a single dose of glycerol 50% at a dose of 10 ml/kg significantly raised serum levels of creatinine and BUN compared to the control group (*P*<0.001). However when administered with glycerol for 4 days, thymoquinone at doses of 1, 2.5, and 5 mg/kg significantly decreased the levels of creatinine and BUN in renal tissue compared to the glycerol group (*P*<0.001). Additionally, the creatinine and BUN levels did not significantly change when thymoquinone alone was injected at a dose of 5 mg/kg compared to the control group (Figure 1A and 1B).

***Effects of thymoquinone on glycerol-induced renal histopathology injury***

**Figure 2:**
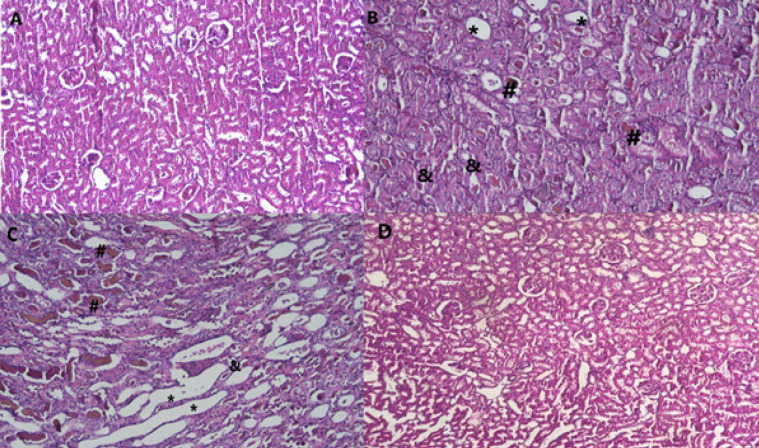
Effect of glycerol 50% (10 ml/kg) and thymoquinone (1, 2.5, and 5 mg/kg) on kidney tissue in Wistar rats

Figure 2 and Table 2 demonstrate that, in comparison to the control group, a single dose injection of glycerol 50% (10 ml/kg) increased kidney tissue damage, including substantial tubular necrosis, protein cast formation, and glomerular atrophy. However, compared to the control group, the administration of thymoquinone 1 and 2.5 mg/kg, along with glycerol did not result in a significant reduction in tubular necrosis, protein cast formation, and glomerular atrophy. In comparison to the glycerol group, the co-administration of thymoquinone at doses of 5 mg/kg, with glycerol significantly reduced tubular necrosis, protein cast formation, or glomerular atrophy. Additionally, rats that received thymoquinone (5 mg/kg) alone showed no apparent variation in tissue histology from the control group. 

***Effect of thymoquinone on glycerol-induced lipid peroxidation in kidney tissues***

**Figure 3:**
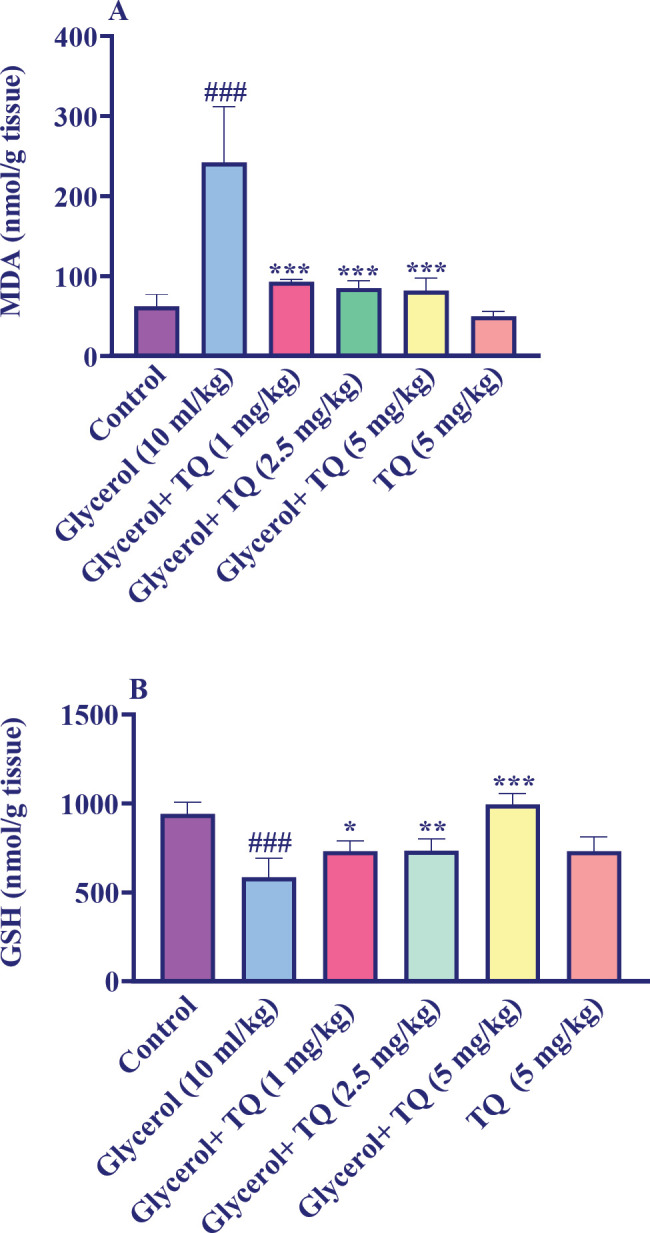
Effect of glycerol 50% (10 ml/kg) and thymoquinone (1, 2.5, and 5 mg/kg) A: MDA and B: GSH levels in the kidney tissue in Wistar rats

The findings showed that kidney tissue MDA level significantly increased after receiving a single dosage of glycerol 50% (10 ml/kg) compared to the control group (*P*<0.001). In contrast, when administered with glycerol, thymoquinone at doses of 1, 2.5, and 5 mg/kg significantly decreased the MDA level (*P*<0.001). Furthermore, the MDA content did not markedly alter when thymoquinone was injected alone at a dose of 5 mg/kg compared to the control group (Figure 3A).

***Effect of thymoquinone on glycerol-induced glutathione depletion in kidney tissues***

According to our research, as shown in Figure 3B, the administration of glycerol 50% 10 ml/kg (single dose) significantly decreased the amount of kidney GSH in comparison to the control group. In comparison to the glycerol group, the co-administration of thymoquinone at doses of 1 mg/kg (*P*<0.05), 2.5 mg/kg (*P*<0.01), and 5 mg/kg (*P*<0.001) significantly increased GSH levels. Notably, there was no obvious difference between the thymoquinone (5 mg/kg) alone group and the control group.

***Effect of glycerol and thymoquinone on TNF-α and NGAL levels in kidney tissues***

The optimal thymoquinone dose, 5 mg/kg, for western blotting was determined based on results obtained from biochemical (Cr and BUN) and oxidative stress factor (MDA and GSH) tests.

**Figure 4:**
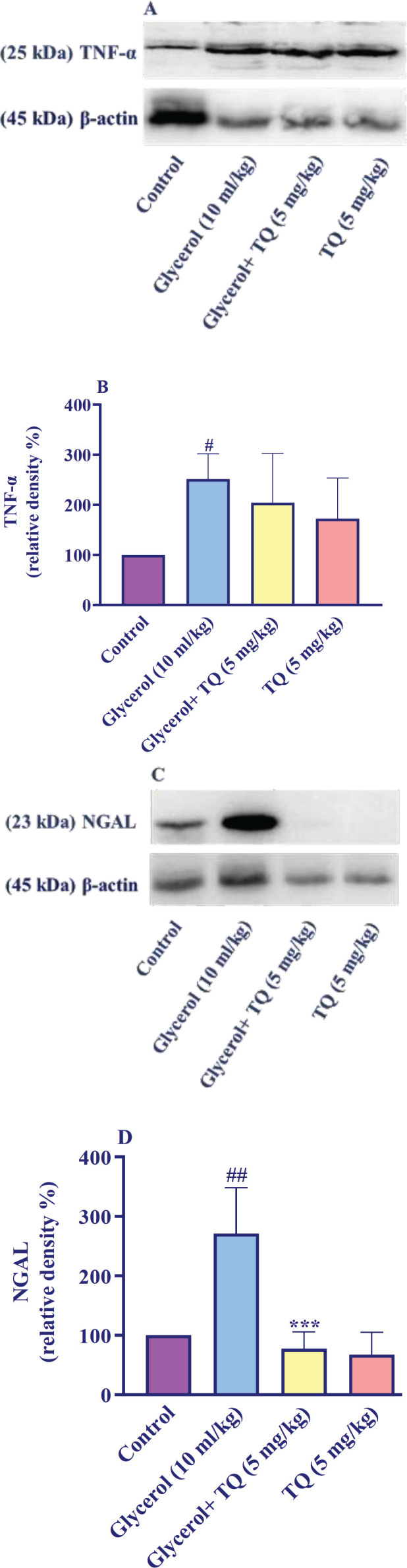
Effect of glycerol 50% (10 ml/kg) and thymoquinone (5 mg/kg) on TNF-α and NGAL levels in kidney tissues in Wistar rats

Comparing the rats that received glycerol with the control group, it showed a statistically significant increase (*P*<0.05) in the TNF-α level. However, when thymoquinone was administered intraperitoneally at a dose of 5 mg/kg for 4 days in conjunction with glycerol, there was no significant alteration in the TNF-α level in the kidney tissue when compared to the glycerol group. Furthermore, administering thymoquinone alone at a dose of 5 mg/kg did not result in any significant alterations of the TNF-α level in the kidney tissue in comparison to the control group (Figure 4A, B). 

Statistical analysis revealed a significant increase in the level of NGAL in the kidney tissue of all rats injected with glycerol compared to the control group (*P*<0.01). However, when thymoquinone was co-administered with glycerol at a dose of 5 mg/kg, the level of NGAL in the kidney tissue was significantly reduced compared to rats that received glycerol alone (*P*<0.001). Besides, administering thymoquinone alone at a dose of 5 mg/kg did not result in any significant changes in NGAL level in the kidney tissue compared to the control group (Figure 4C, D).

**Table 1:** Study design for evaluation acute kidney injury in Wistar rats

**Groups**	**Treatment**	**Dosage**	**Route of exposure**	**Duration (Day)**
1	Control	-	i.m. + IP	4
2	Glycerol	50 ml/kg	i.m.	1
3	Glycerol+ TQ	50 ml/kg + 1 mg/kg	i.m. + IP	4
4	Glycerol+ TQ	50 ml/kg + 2.5 mg/kg	i.m.	4
5	Glycerol+ TQ	50 ml/kg + 5 mg/kg	i.m. + IP	4
6	TQ	5 mg/kg	i.p	4

TQ: Thymoquinone ; IP: Intraperioneal; IM: Intramuscular

Data are expressed as mean±SD, (n=6). Statistical difference was examined using the ANOVA test and a Tukey-Kramer post-test. ### *P*<0.001 vs control group, *** *P*<0.001 vs glycerol group

TQ: Thymoquinone

Tissue sections were stained with hematoxylin and eosin (H&E) and observed under a light microscope. Group A: represents the control group, B: glycerol, C: glycerol+ thymoquinone (1, 2.5, and 5 mg/kg), and D: thymoquinone (5 mg/kg). * indicates tubular necrosis, # indicates protein cast formation, and & indicates glomerular atrophy. (hematoxylin and eosin staining, magnification 400x)

**Table 2:** Histopathological alterations in different Wistar rats groups

**Groups**	**Treatment**	**Dosage**	**Glomerular atrophy**	**Myoglobin cast**	**tubular necrosis**
1	Control	-	**-**	**-**	**-**
2	Glycerol	50 ml/kg	**++++**	**++++**	**++++**
3	Glycerol+ TQ	50 ml/kg + 1 mg/kg	**+++**	**+++**	**+++**
4	Glycerol+ TQ	50 ml/kg + 2.5 mg/kg	**+++**	**+++**	**+++**
5	Glycerol+ TQ	50 ml/kg + 5 mg/kg	**++**	**++**	**++**
6	TQ	5 mg/kg	**-**	**-**	**-**

(-) Normal (+) Mild damage (++) Moderate damage (+++) Severe damage (++++) Extensive damage

Data are expressed as mean±SD (n=6). Statistical difference was examined using the ANOVA test and a Tukey-Kramer post-test. ### *P*<0.001 vs control group, *** *P*<0.001 vs glycerol group.

TQ: Thymoquinone

A, C: Show immunoblot bands of the western blotting analysis and B, D: Display quantitative presentation of the immunoblots. Data were analyzed by one-way ANOVA following Tukey’s for multiple comparisons. # *P*<0.05 and ##* P*<0.01 vs control group, *** *P*<0.001 vs glycerol group.

TQ: Thymoquinone

## Discussion

The objective of the current study was to investigate the potential renoprotective effects of thymoquinone on AKI induced by rhabdomyolysis in rats. The results of this study revealed that the administration of glycerol 50% (10 ml/kg) caused a significant increase in levels of serum creatinine, BUN, as well as MDA, TNF-α, and NGAL amounts in kidney tissues, while reducing GSH levels and causing renal tubular necrosis, glomerular atrophy, and myoglobin cast in kidney tissues. However, co-treatment with thymoquinone at doses of 1, 2.5, and 5 mg/kg resulted in reduced levels of creatinine, BUN, MDA, and histopathological alterations as well as increased GSH levels. Notably, concurrent administration of glycerol and thymoquinone (5 mg/kg) did not have a significant effect on TNF-α levels but decreased NGAL protein levels.

The injection of glycerol is a widely used method for inducing an AKI model, as it causes skeletal muscle degeneration and the release of its contents into circulation, resulting in a myoglobinuric state similar to clinical rhabdomyolysis (4). The presence of renal impairment was indicated by significant increases in serum levels of urea and creatinine after glycerol 50% (10 ml/kg) injection (4, 30). The same results have been achieved in our study and a single glycerol 50% (10 ml/kg) injection led to elevated serum creatinine and BUN levels. Remarkably, the co-administration of glycerol and thymoquinone (1, 2.5, and 5 mg/kg, 4 days, IP) was found to reverse these alterations. Similarly, the administration of thymoquinone (10 mg/kg, 8 weeks) lowered serum creatinine, urea, and creatinine clearance in rats with streptozotocin-induced diabetic nephropathy (31). Also, thymoquinone (50 mg/kg, 4 weeks, PO), amended gentamicin-induced renal dysfunction in rats by reducing serum levels of creatinine and urea (32).

The histopathological assessment of renal tissue supported the earlier findings, as it revealed renal tubular necrosis, glomerular atrophy, and myoglobin cast in kidney tissues. In line with our obtained data, another study has also reported tubular ischemia, vacuolation, and subsequent tubular necrosis and glomerular injury following glycerol 50% (10 ml/kg) injection (4). Our study found that the administration of thymoquinone markedly ameliorated the observed histopathological alterations. Also, a previous study reported that thymoquinone (50 mg/kg, 4 weeks, PO), reduced endothelial proliferation and mesangial hypercellularity in rats with gentamicin-induced renal dysfunction (32). Additionally, thymoquinone (20 mg/kg, every other day for 21 days, PO) was found to reverse inflammatory cell infiltration, dilated hyperemic veins, glomerular atrophy, and proximal tubular necrosis in rats with gentamicin-induced renal disorder (33). 

The pathogenesis of rhabdomyolysis induced-AKI has been linked to the production of oxygen free radicals derived from myoglobin. Consistent with this hypothesis, studies have shown that oxidative stress plays a critical role in rhabdomyolysis induced-AKI by demonstrating an elevation in the renal concentration of markers such as 8-OHdG (8-hydroxy-2’-deoxyguanosine) and MDA, which is indicative of lipid peroxidation (34, 35). Additionally, a decrease in renal levels of the antioxidant enzyme superoxide dismutase (SOD) has also been observed (36). Consistent with earlier research, our findings demonstrated that a single intramuscular injection of 50% glycerol (10 ml/kg) led to a significant increase in MDA levels and a decrease in GSH levels in kidney tissue. However, when glycerol and thymoquinone (1, 2.5, and 5 mg/kg, 4 days, IP) were administered concurrently, MDA levels were significantly reduced and GSH levels were enhanced in kidney tissue. Our findings are consistent with a previous study which demonstrated that thymoquinone (50 mg/kg, 4 weeks, PO) improved gentamicin-induced renal dysfunction in rats by reducing tissue MDA levels and increasing levels of glutathione peroxidase-1 and SOD (32). Furthermore, the renoprotective effects of thymoquinone (25 and 50 mg/kg, 3 months, PO) against chronic toxicity induced by sodium nitrite in rats have also been reported (37). In a study conducted on rats, thymoquinone (10 mg/kg, 15 days, PO) showed protective effects against oxidative stress induced by arsenic in the kidney by reducing MDA levels and increasing the levels of SOD, catalase, and glutathione peroxidase in kidney tissue (38).

The involvement of TNF-α in injuries following rhabdomyolysis is significant. The primary contributor to acute tubular injury in the glycerol model is TNF-α-mediated necroptosis (35). He *et al*. have additionally reported that the administration of glycerol 50% (8 ml/kg) led to an up-regulation of TNF-α messenger ribonucleic acid (mRNA) expression in the kidneys of mice (39). Our research team has also observed an increase in TNF-α levels in kidney tissue following glycerol injection. Furthermore, a four-day treatment with thymoquinone (5 mg/kg) did not result in a significant reduction of TNF-α levels. In contrast to our observations, prior research has reported that thymoquinone (10 mg/kg, 10 days, PO) reduced TNF-α levels in rats with ischemia-reperfusion injury (17) and that thymoquinone (50 mg/kg, 2 weeks, PO) decreased TNF-α levels in mice with sepsis-induced AKI (40). The inconsistency between our results and earlier studies could be attributed to variations in the dosage and duration of thymoquinone treatment, as well as the severity of symptoms in various experimental models of renal disorders.

Besides, NGAL, a member of the lipocalin superfamily, has been identified not only as a stress protein but also as an early biomarker for the diagnosis of AKI (4, 36). Our findings are consistent with previous studies, as we observed a remarkable elevation in NGAL levels following intramuscular injection of glycerol 50% (10 ml/kg). However, we also found that co-administration of glycerol and thymoquinone (5 mg/kg) resulted in a notable reduction of NGAL levels when compared to the glycerol group. Consistent with our findings, in the previous study, oral administration of thymoquinone (10 mg/kg, 10 days) increased renal NGAL levels following ischemia-reperfusion injury in rats (17). Furthermore, oral administration of thymoquinone (20 mg/kg, 5 days) significantly reversed the enhancement in NGAL amounts in rats with combined cisplatin and diesel exhaust particles induced renal toxicity (41). 

## Conclussion

In summary, the present study demonstrates that thymoquinone seems to have renoprotective effects on AKI induced by rhabdomyolysis. Thymoquinone co-treatment resulted in reduced levels of creatinine, BUN, MDA, histopathological alterations, and NGAL, as well as increased GSH levels. The discrepancy in the renal TNF-α levels and histopathological results between our study and previous studies may be due to differences in the dosage and duration of thymoquinone treatment and the severity of symptoms in different experimental models of renal disorders. Thymoquinone seems to be a promising agent in ameliorating rhabdomyolysis induced-AKI. However, further studies are needed to elucidate its mechanism of action in AKI induced by rhabdomyolysis.

## Authors’ Contributions

H H and S M conceived the study and supervised the group throughout the study. A KH supervised the thesis. A H conducted the experiments and analyzed the data. A H and M GR wrote the manuscript collaboratively and generated the statistical graphs. T A and S N assisted with the experiments. A J performed the pathology tests. All authors reviewed and approved the final manuscript.

## Conflicts of Interest

 The authors declare that they have no conflicts of interest.
